# Ticagrelor Can Regulate the Ion Channel Characteristics of Superior Cervical Ganglion Neurons after Myocardial Infarction

**DOI:** 10.3390/jcdd10020071

**Published:** 2023-02-06

**Authors:** Lijun Cheng, Lin Yu, Xiaoping Zhan, Gary Tse, Tong Liu, Huaying Fu, Guangping Li

**Affiliations:** 1 Tianjin Key Laboratory of Ionic-Molecular Function of Cardiovascular Disease, Department of Cardiology, Tianjin Institute of Cardiology, The Second Hospital of Tianjin Medical University, Tianjin 300211, China; 2 School of Nursing and Health Studies, Hong Kong Metropolitan University, Hong Kong, China

**Keywords:** superior cervical ganglion, myocardial infarction, P2Y12 receptor antagonist, ion channel

## Abstract

Background: The superior cervical ganglion (SCG) plays a key role in cardiovascular diseases. The aim of this study was to determine the changes in the ion channel characteristics of the SCG following myocardial infarction (MI) and the role of pretreatment with the P2Y12 receptor antagonist ticagrelor (TIC). Methods: A total of 18 male rabbits were randomly divided into a control group, MI group, and P2Y12 receptor antagonist (TIC) group (abbreviated as the TIC group). Rabbit MI was performed via two abdominal subcutaneous injections of 150 mg·kg^−1^·d^−1^ of isoproterenol (ISO) with an interval of 24 h. TIC pretreatment at 20 mg·kg^−1^·d^−1^ was administered via gavage for two consecutive days. The cardiac function of each group was evaluated with echocardiography. ADP receptor P2Y12 expressions in SCGs were determined using RT-PCR and immunofluorescence staining. Ion channel characteristics of SCG neurons were measured using a whole-cell patch clamp. Intracellular calcium concentrations for SCG neurons were measured using confocal microscopy. Results: Cardiac function was reduced in the rabbits of the MI group, the sympathetic nerve activity of SCGs was increased, and the current amplitude of the neuron ion channel was increased. MI led to alterations in the activation and inactivation characteristics of *I*_Na_ channels accompanied by increased expression of P2Y12 in SCGs. Most of these abnormalities were prevented by TIC pretreatment in the TIC group. Conclusions: TIC pretreatment could attenuate the increase in P2Y12 expression in SCGs and the changes to the ion channel characteristics of SCG neurons after MI. This may be the mechanism underlying the cardiac protective effects of TIC.

## 1. Introduction

Myocardial infarction (MI), as one of the common cardiovascular diseases, is a significant problem for middle-aged and elderly individuals. Cardiac sympathetic nerves are over-excited after MI [1,2], and the accumulation of neurotransmitters is an important cause and mechanism of arrhythmia after MI [3,4]. The superior cervical ganglion (SCG) is an important extracardiac ganglion that participates in the regulation of heart function. Previous studies have demonstrated that many components of SCGs are involved in the regulation of cardiac function after MI [5,6]. Our previous results showed that the electrophysiological levels of extracardiac ganglion neurons increased significantly after myocardial ischemia and infarction [7,8,9,10]. These changes in SCGs were closely related to the occurrence of ventricular arrhythmias after MI. Therefore, attenuating the activity of SCGs in MI patients can prevent abnormal cardiac electrophysiological function. 

Ion channels mediate electrical activity in neuronal cells, and these channels for SCG neurons provide the electrophysiological basis of sympathetic nerve excitability. Our prior studies have shown that, after myocardial ischemia, the characteristics of the ion channels of the SCG neurons changed significantly, and the channel current amplitude increased [8]. Reducing changes to the electrical properties of ion channels helped to reduce the increase in sympathetic nerve activity after MI. 

P2Y12, a P2Y metabotropic G-protein-coupled purinergic receptor, is expressed in the sympathetic ganglia and can regulate neuronal function [11]. P2Y12 was found to be involved in the enhanced excitatory synaptic responses in the substantia gelatinosa neurons after nerve injury [12]. After MI, the expression of P2Y12 receptors in some sympathetic ganglia increased significantly [11]. Moreover, P2Y12 affects ion channel characteristics [13,14]. P2Y12 receptor inhibitors can improve ion channel function [15] and protect against ischemia-induced neural injury [16]. Therefore, P2Y12 may be involved in the process of changing the electrical properties of SCG neurons’ ion channels after MI. The use of P2Y12 receptor inhibitors may be beneficial for the recovery of sympathetic nerves after MI, but the specific mechanism is not clear. The aim of this study was to investigate the role of the P2Y12 receptor antagonist ticagrelor (TIC) in electrophysiological remodeling of SCG neurons in a rabbit model of MI. 

## 2. Materials and Methods

### 2.1. Animals and Experimental Design

This study was approved by the Animal Ethical and Welfare Committee of the Chinese Academy Medical Sciences Institute of Radiation Medicine. The animals’ use, their groupings, and the MI preparation methods for experimental animals are briefly introduced as follows: 18 male rabbits weighing 300–500 g (Tianjin Yuda Experimental Animal Co., Ltd., Tianjin, China) were randomly and equally divided into a control group, MI group, and P2Y12 receptor antagonist (TIC) group (abbreviated as the TIC group). Rabbit MI was performed via two abdominal subcutaneous injections of 150 mg·kg^−1^·d^−1^ of isoproterenol (ISO) with an interval of 24 h. The successful establishment of the MI model was verified in our previous study [7]. Rabbits in the TIC groups were administered 20 mg·kg^−1^·d^−1^ TIC (on two consecutive days) via gavage, then MI was induced via ISO. Rabbits in the control group were administered a normal saline injection subcutaneously. After 24 h of treatment, SCG tissues and blood were collected from the three groups of rabbits for the following experiments. The successful establishment of the MI model was determined through the assessment of myocardial enzymes (creatine kinase (CK) and creatine kinase isoenzyme (CKMB)), electrocardiograms (ECGs), and echocardiography recordings. 

### 2.2. Echocardiography

After 24 h of the intervention, rabbits in each group were anesthetized with 3% pentobarbital sodium to perform transthoracic echocardiography. Echocardiography parameters, including left atrial diameter (LAD), left ventricular end-diastolic dimension (LVDD), left ventricular ejection fraction (LVEF), and fractional shortening (FS), were recorded using a small-animal ultrasound system (Vevo 2100, VisualSonics, Toronto, ON, Canada). 

### 2.3. Preparation and Electrophysiological Recordings of SG Neurons

The methods for the SCG neuron acquisition and whole-cell patch-clamp recording were described in our previous reports [7,8,9,10]. In short, SCG slices were cut, digested in the enzyme solution (50–60 min, 37 °C), and dispersed gently with glass tubes into a single neuron. For the enzyme solution, 0.6–0.7 g/L pronase E (Roche, Basel, Switzerland), 1.7–1.8 g/L collagenase type II (Worthington, Lakewood, CO, USA), and 7.0–8.0 g/L bovine serum albumin (Roche, Basel, Switzerland) were added to the incubation solution (NaCl 130 mmol/L, MgCl_2_ 1 mmol/L, KCl 5 mmol/L, glucose 10 mmol/L, CaCl_2_ 2 mmol/L, NaH_2_PO_4_ 1.5 mmol/L, NaHCO_3_ 25 mmol/L, and HEPES 10 mmol/L). Here, we mainly recorded the characteristics of the delayed rectifier potassium channel current (*I*_K_), sodium channel current (*I*_Na_), and N-type calcium channel current (*I*_Ca_). The data was recorded using Axopach 200B patch-clamp systems and analyzed using Clampex 10.2 software (Axon, Scottsdale, AZ, USA). 

### 2.4. Intracellular Calcium Measurement in SCG Neurons

The method for recording the intracellular calcium concentrations of SCG neurons was described in detail in our previous studies [7,8]. The SCG neurons were loaded in incubation solution including 3 μM Fluo 4-AM and Pluronic F-127 (<0.05%) (40 min, 37 °C). After that, the change in the fluorescence intensity in SCG neurons was monitored using confocal microscopy (FV1000, Olympus, Tokyo, Japan). F/F_0_ was used to reflect intracellular calcium concentrations, where F_0_ and F are the fluorescence intensity before and after adding 60 mmol/L KCl, respectively. 

### 2.5. Immunofluorescence Staining

SCG tissues were fixed in 4% paraformaldehyde, embedded in paraffin, sectioned into 5 μm slices, and incubated with anti-tyrosine hydroxylase (TH, 1:300, Boster, Wuhan, China) and anti-P2Y12 (1:200, Bioss, Beijing, China) overnight at 4 °C. After washes with PBS, SCG tissue slices were incubated with CoraLite594-conjugated Goat Anti-Rabbit IgG (1:200, Proteintech, Wuhan, China) and CoraLite488-conjugated Affinipure Goat Anti-Mouse IgG (1:200, Proteintech, Wuhan, China) secondary antibodies. In order to identify the nucleus, the slices were counterstained with 4′,6-diamidino-2-phenylindole (DAPI, Solarbio, Beijing, China). Then, the fluorescence intensity of the slices was detected using a confocal microscope (FV1000). 

### 2.6. RT-PCR

Briefly, total RNA extraction and cDNA synthesis were completed using a kit (Tiangen, Beijing, China) following the manufacturer’s instructions. RT-PCR was performed with a Quant Gene 9600 System (Bioer Technology, Hangzhou, China). Relative gene expression was calculated with the 2-ΔΔCT method. All values obtained with the P2Y12 primers were normalized to the values obtained with the GAPDH primers. The primer sequences of GAPDH were as follows: (forward) TCGTGGATGACCTTGGCC and (reverse) GATGCTGGTGCCGAGTAC. The primer sequences of P2Y12 were as follows: (forward) ACCAGTTTGGAACCGCTAAA and (reverse) GTAGGCCCACATCAAATGCT. 

### 2.7. ELISA

Blood samples were collected for norepinephrine (NE) determination 24 h after MI and TIC intervention. Serum was collected and stored at −80 °C. We used a commercial NE ELISA kit (Cloud-Clone Corp, Wuhan, China) to measure the NE concentration in rabbit serum. 

### 2.8. Statistical Analysis

The variables are presented as means ± standard deviation (SD). The comparison between the groups was analyzed with one-way ANOVA. An LSD *t*-test was used for post hoc analysis. *p* < 0.05 was considered to be statistically significant. Data were analyzed with Origin 6.0 (OriginLab, Northampton, MA, USA), Clampex 10.2, and SPSS 17.0 software (SPSS Inc., Chicago, IL, USA).

## 3. Results

### 3.1. MI Model Validation and Changes in Cardiac Function after MI and TIC

**Figure 1:**
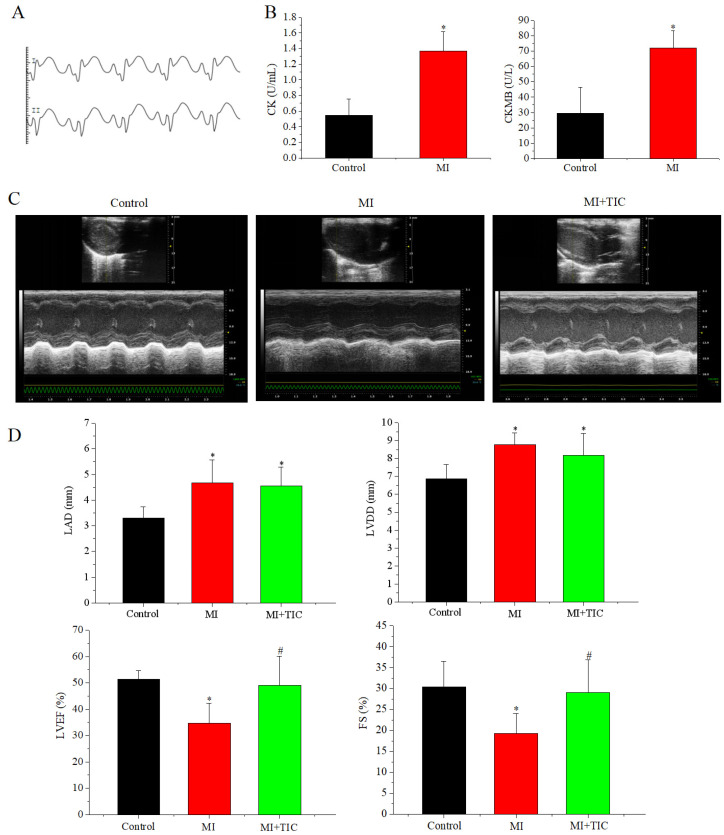
MI model validation and changes in cardiac function after MI and TIC. (**A**) ECG of MI group; (**B**) CK and CKMB in serum in control and MI groups; (**C**) sample echocardiography images for the three groups; (**D**) histograms of LAD, LVDD, LVEF, and FS in the three groups. MI, myocardiac infarction; TIC, ticagrelor; LAD, left atrial diameter; LVDD, left ventricular end-diastolic dimension; LVEF, left ventricular ejection fraction; FS, fractional shortening. Each point represents the mean ± SD, * *p* < 0.05 compared with the control group. ^#^ *p* < 0.05 compared with the MI group.

MI was validated by (a) increased levels of myocardial enzymes, (b) ST segment elevation in ECGs, and (c) reduction in cardiac function (Figure 1A–D). The effects of MI and TIC on cardiac function were detected by echocardiography. Compared with the control group, the cardiac chamber dilation was decreased and left ventricle function reduced in the MI group, which manifested as increasing LAD and LVDD and decreasing LVEF and FS (*n* = 6, *p* < 0.05; Figure 1C,D). These changes were mostly reversed by the TIC intervention. Thus, although TIC did not completely restore cardiac function after MI, it improved cardiac function. 

### 3.2. P2Y12 Expression in SCG and NE content in Serum after MI and TIC

**Figure 2:**
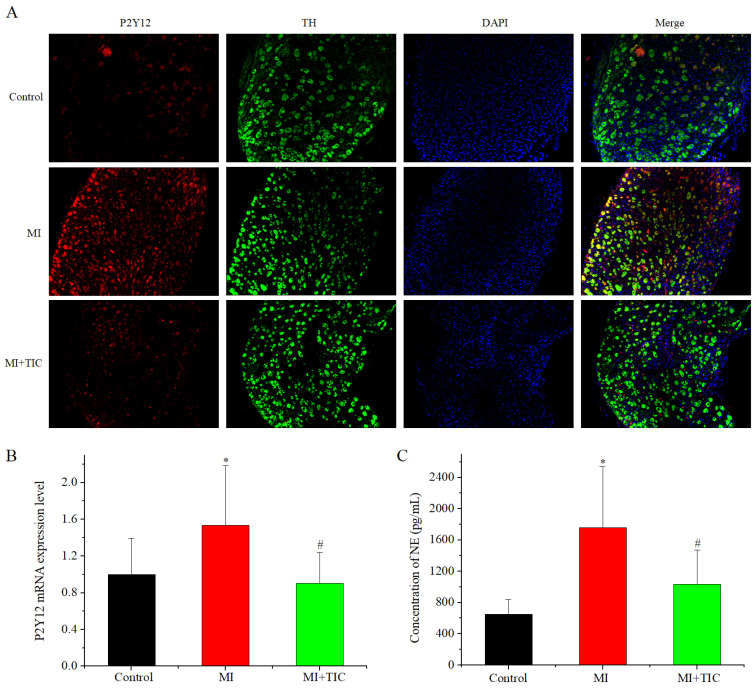
Expression of P2Y12 in SCGs after MI and TIC. (**A**) P2Y12 and TH distributions for the three groups were determined by immunofluorescence staining; (**B**) P2Y12 expression in the three groups was determined by RT-PCR; (**C**) the NE content in the serum of the three groups. SCG, superior cervical ganglion; MI, myocardiac infarction; TIC, ticagrelor; TH, tyrosine hydroxylase. Each point represents the mean ± SD, * *p* < 0.05 compared with the control group. ^#^ *p* < 0.05 compared with the MI group.

First, we examined the effects of MI and TIC treatment on P2Y12 expression in SCGs using immunofluorescence staining and RT-PCR. Double staining with antibodies against TH and P2Y12 was applied to SCG neurons. The results showed that P2Y12 was mainly expressed in the TH neurons in the SCGs of the three groups. Immunofluorescence and RT-PCR results showed that P2Y12 levels in SCGs were significantly higher in the MI group compared to the control group (*n* = 5, *p* < 0.05) and significantly lower in the TIC group compared to the MI group (*n* = 5, *p* < 0.05) (Figure 2A,B). As sympathetic activity is directly related to neurotransmitter release, we examined the effects of TIC and MI on the NE content in serum. The ELISA results showed that NE content in serum was significantly increased in MI-group rabbits compared to those in the control group (*n* = 6, *p* < 0.05) and significantly lower in the TIC group compared to the MI group (*n* = 6, *p* < 0.05) (Figure 2C). It can be seen that TIC treatment can significantly improve P2Y12 expression in the SCGs and NE content in serum after MI. Next, we examined the effects of MI and TIC on electrophysiological properties and intracellular calcium concentrations in SCG neurons. 

### 3.3. Effects of MI and TIC on Activation Kinetics of I_K_

**Figure 3:**
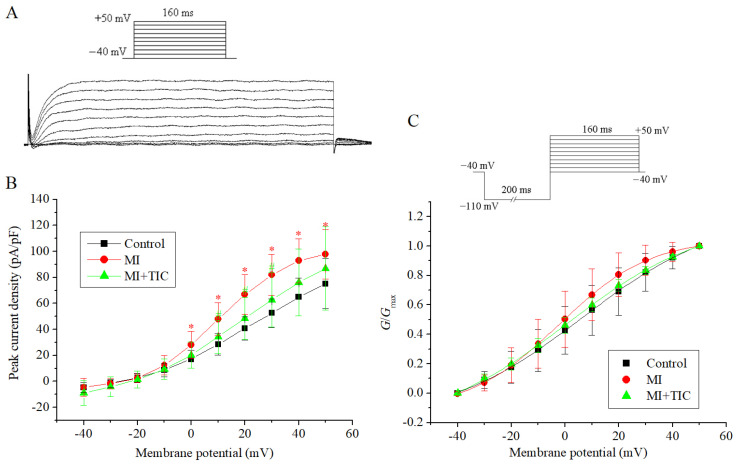
Effects of MI and TIC on *I*_K_ characteristics. (**A**) The patch-clamp protocol and typical examples of *I*_K_; (**B**) I-V curves for *I*_K_ for the three groups; (**C**) the patch-clamp protocol and activation curves for *I*_K_ in the three groups. MI, myocardiac infarction; TIC, ticagrelor. Each point represents the mean ± SD, * *p* < 0.05 compared with the control group. ^#^ *p* < 0.05 compared with the MI group.

The patch-clamp protocol and the drawing of the curves (current-voltage (I-V) curves and activation curves) for *I*_K_ were carried out with reference to our previous study [7,8]. I-V curves were drawn by plotting the peak current density (the x-axis represented the different test membrane potentials; the y-axis represented the maximum current amplitude/neuron membrane capacitance). The patch-clamp protocol, curve sample, and I-V curves for *I_K_* are shown in Figure 3A,B. As seen in the I-V curve, the peak current density of *I*_K_ was increased (97.76 ± 18.91 vs. 75.15 ± 19.14 pA/pF, *n* = 10, *p* < 0.05) in the MI group. TIC pretreatment slightly reduced the current amplitude of *I*_K_ (86.99 ± 32.02 pA/pF), but there was no significant difference with the MI group (*n* = 10, *p >* 0.05). 

The patch-clamp protocol and the activation curves for *I*_K_ are shown in Figure 3C. The current amplitude with different membrane potentials was converted into conductance using the equation: *G* = *I*/(*V* − *V*_rev_), where *V*_rev_ is the reversal potential and *V* is the membrane potential. The activation curves were fitted using the Boltzmann equation: *G*/*G*_max_ = 1/{1 + exp[(*V*_1/2_ − *V*)/*k*]}, where *G*_max_ is the maximum conductance, *V*_1/2_ is the potential at half-activation, and *k* is the slope factor of the curves. According to the activation curve-fitting results, the *V*_1/2_ values for the three groups were 6.35 ± 0.64 mV, −2.35 ± 0.38 mV, and 0.68 ± 0.68 mV (*n =* 10, *p >* 0.05). The *k* values for the three groups were 25.79 ± 1.11, 16.99 ± 0.43, and 25.51 ± 1.08 (*n =* 10, *p >* 0.05). A comparison showed that there were no significant differences in the activation curves for the three groups (*n =* 10, *p >* 0.05). Therefore, TIC could improve the current amplitude of *I*_K_, but neither MI nor TIC had an effect on the activation curve of *I*_K_. 

### 3.4. Effects of MI and TIC on Activation Kinetics of I_Na_

**Figure 4:**
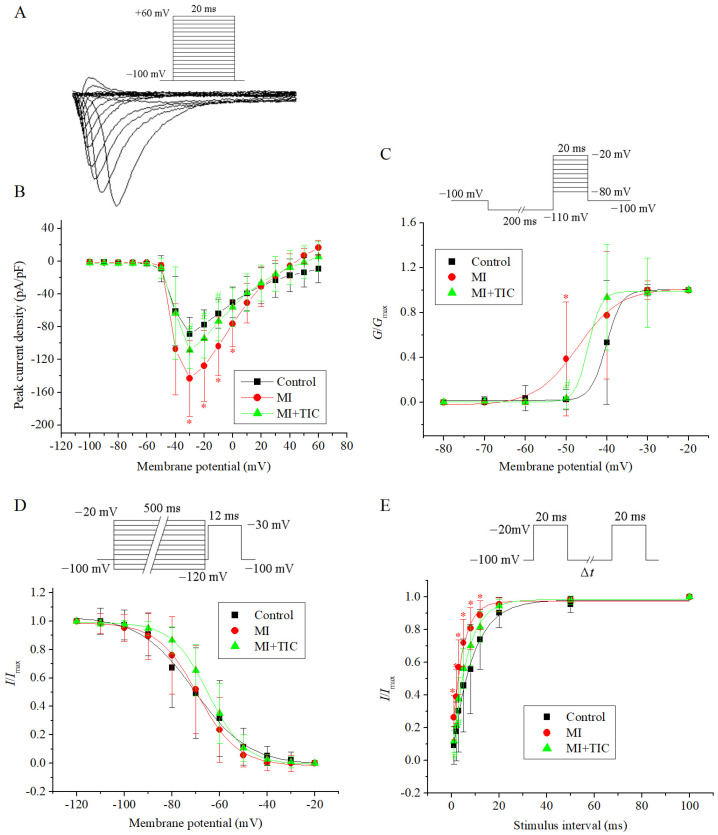
Effects of MI and TIC on *I*_Na_ characteristics. (**A**) The patch-clamp protocol and typical examples of *I*_Na_; (**B**) I-V curves for *I*_Na_ for the three groups; (**C**) the patch-clamp protocol and activation curves for *I*_Na_ for the three groups; (**D**) the patch-clamp protocol and inactivation curves for *I*_Na_ for the three groups; (**E**) the patch-clamp protocol and recovery-from-inactivation curves for *I*_Na_ for the three groups. MI, myocardiac infarction; TIC, ticagrelor. Each point represents the mean ± SD, * *p* < 0.05 compared with the control group. ^#^ *p* < 0.05 compared with the MI group.

The patch-clamp protocol and the drawing of the curves (I-V curves, the activation curves, the inactivation curves, and the recovery curves) for *I*_Na_ were carried out with reference to our previous study [7,8]. The patch-clamp protocol, curve sample, and I-V curves for *I*_Na_ are shown in Figure 4A,B. I-V curves were drawn using the aforementioned method. The I-V curve showed that the peak amplitude of the *I*_Na_ density was increased (−143.30 ± 46.11 vs. −88.96 ± 20.65 pA/pF, *p* < 0.05) in the MI group. TIC pretreatment could reduce the current amplitude of *I*_Na_ (−108.52 ± 21.88 pA/pF, *n =* 10, *p* < 0.05). 

The patch-clamp protocol and activation curves for *I*_Na_ for the three groups are shown in Figure 4C. The activation curves were drawn using the aforementioned method. The fitting results for the activation curve showed that the *V*_1/2_ values for the three groups were −40.18 ± 0.27 mV, −47.11 ± 0.64 mV, and −44.59 ± 0.93 mV (*n =* 10, *p >* 0.05). The *k* values were 1.88 ± 1.78, 5.18 ± 0.57, and 1.61 ± 0.30 (*n =* 10, *p* < 0.05). The activation curves for the MI group were shifted toward negative potential compared to those of the control group, and the *k* values in the activation curves were increased (*n =* 10, *p* < 0.05). However, these changes were partially reversed in the TIC group. 

The patch-clamp protocol and inactivation curves for *I*_Na_ for the three groups are shown in Figure 4D. The inactivation curves were fitted with the Boltzmann equation: *I*/*I*_max_ = 1/{1 + exp[(*V* − *V*_1/2_)/*k*]}, where *I*_max_ is the maximum current amplitude, *V*_1/2_ is the potential at half-inactivation, and *k* is the slope factor. From the fitting results for the inactivation curve, the *V*_1/2_ values for the three groups were −70.63 ± 0.72 mV, −69.34 ± 0.55 mV, and −64.77 ± 0.51 mV (*n =* 10, *p >* 0.05). The *k* values were 11.36 ± 0.71, 8.45 ± 0.50, and 7.69 ± 0.46 (*n =* 10, *p >* 0.05). Compared to the control group, the inactivation curves for the MI group and TIC group did not change significantly (*n =* 10, *p* > 0.05). 

The patch-clamp protocol and the recovery curves for *I*_Na_ for the three groups are shown in Figure 4E. The intervals of the double pulses were 1 ms, 2 ms, 3 ms, 5 ms, 8 ms, 12 ms, 20 ms, 50 ms, and 100 ms, respectively. The recovery curves for *I*_Na_ were fitted with the exponential equation: *I*/*I*_max_ = 1 − exp(−*t*/*τ*), where *I*_max_ is the maximum current amplitude, *t* is the recovery time, and *τ* is the time constant for channel recovery. The fitting results for the recovery curve showed that the *τ* values of the three groups were 8.45 ± 0.60 ms, 4.30 ± 0.39 ms, and 6.09 ± 0.44 ms (*n =* 10, *p* < 0.05). The recovery curves for the MI group were shifted toward negative potential compared to those of the control group, and *τ* values were reduced (*n =* 10, *p* < 0.05). These changes were mostly reversed in the TIC group (*n =* 10, *p >* 0.05). Therefore, TIC could improve the changes to *I*_Na_ characteristics after MI, including the current amplitude, activation, and recovery curve.

### 3.5. Effects of MI and TIC on I_Ca_ and Intracellular Calcium Concentration 

**Figure 5:**
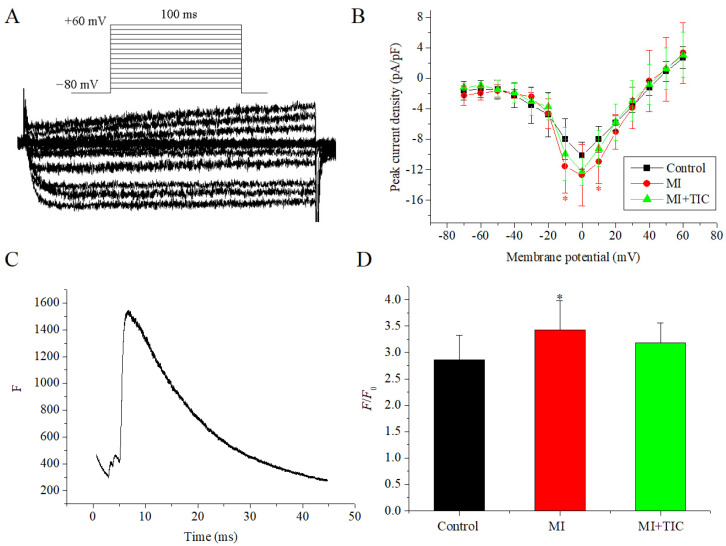
Effects of MI and TIC on *I*_Ca_ and intracellular calcium concentration. (**A**) The patch-clamp protocol and typical examples of *I*_Ca_; (**B**) I-V curves for *I*_Ca_ for the three groups; (**C**) representative recordings of fluorescence curves for intracellular calcium concentration. (**D**) Ratio of peak to resting fluorescence (F/F_0_ ratios) for the three groups. MI, myocardiac infarction; TIC, ticagrelor. Each point represents the mean ± SD, * *p* < 0.05 compared with the control group.

Next, we measured the *I*_Ca_ characteristics and the intracellular calcium concentration of SCG neurons after MI. The patch-clamp protocol, curve sample, and I-V curves for *I*_Ca_ are shown in Figure 5A,B. The I-V curves were drawn using the aforementioned method. The I-V curve showed that the peak amplitude of the *I*_Ca_ density was increased (−12.73 ± 4.04 vs. −10.14 ± 1.79 pA/pF, *n =* 8, *p* > 0.05) in the MI group. This was reduced by TIC pretreatment (−12.23 ± 1.81 pA/pF, *n =* 8, *p* > 0.05). When the voltage was at −10 mV and 10 mV, the peak amplitude of the MI group increased significantly (*p* < 0.05). Although the current amplitude of the TIC group decreased compared to that of the MI group, there was no significant difference between the TIC group and MI group (*p* > 0.05). 

The intraneuronal calcium concentration was obtained using Fluo 4-AM staining and confocal microscopy. Representative recordings of fluorescence curves and F/F_0_ ratios (*n =* 10) are shown in Figure 5C. The F/F_0_ ratio was higher in the MI group compared to that in the control group (3.42 ± 0.55 vs. 2.86 ± 0.47; *p* < 0.05), and TIC pretreatment (3.18 ± 0.37) had no significant effects compared to the control and MI groups (*p* > 0.05). TIC pretreatment could partly attenuate the effects of MI on the current amplitude of the calcium channel and the intracellular calcium concentration. 

## 4. Discussion

In the present study, we investigated the effects of P2Y12 receptor antagonist (TIC) on P2Y12 expression and the electrophysiological characteristics of SCG neurons after MI. After MI, cardiac function and the NE content in serum changed, the expression of P2Y12 protein in SCGs increased, and the characteristics of neuronal ion channels were altered. TIC pretreatment produced reversals of these changes in SCGs. 

### 4.1. Remodeling in SCG Neurons following MI

The sympathetic nervous system (SNS) plays an important role in the regulation of cardiac function after MI [17,18]. MI can lead to excessive excitement of sympathetic nerves, leading to neurotransmitter accumulation [1,2], which is an important reason for the cardiac arrhythmias observed post-MI [3,4]. However, little is known about the molecular mechanisms regulating cardiac sympathetic innervation. The SCG and the stellate ganglion (SG) are important extracardiac ganglia of the heart. As the SG is closer to the heart, there are more studies on the SG. Our previous study showed that not only were the electrophysiological properties of SG neurons significantly altered after myocardial ischemia or infarction but the neuronal electrophysiological properties of the SCG were also significantly altered after MI [7,8,9,10]. In fact, the SCG plays a crucial role in the regulation of cardiac function. Anatomic evidence that the SCG innervates the heart has been reported for a long time, and it is known that postganglionic fibers participate in the formation of the cardiac plexus, innervating myocardial tissues. SCG blockade was also found to improve cardiac fibrosis and cardiac function, and the instability of ventricular electrophysiology was reduced [5]. Many components of SCGs are involved in the regulation of cardiac function after MI. For example, activation of the GABAergic signaling system in SCG sympathetic neurons can suppress sympathetic activity, thereby facilitating cardiac protection and making it a potential target to alleviate ventricular arrhythmias [6]. The expression of the oxytocin receptor in SCGs was enhanced after MI, suggesting that the involvement of the oxytocin receptor in SCGs may contribute to the transmission of sympathetic responses after MI [19]. ATP and the P2X7 receptor in the SCG also participate in sympathoexcitatory transmission after myocardial ischemia injury [20,21]. All these findings indicate that SCGs play important roles in pathophysiology after MI. Our previous results showed that MI led to significantly higher sympathetic neuron activity, which was confirmed by the electrophysiological activity of SCG neurons increasing significantly after myocardial ischemia and infarction [7,8,9]. The experimental results of the present study also confirmed that not only did the NE content in serum increase but the ion channel and intracellular calcium concentration of SCG neurons also changed significantly after MI. These changes reflected the activation of sympathetic nerves after MI, which then induced cardiac electrophysiological instability. Therefore, reducing the activity of SCGs following MI is helpful to prevent dysfunction in the sympathetic nerves and cardiac electrophysiological instability.

### 4.2. Ion Channels in SCGs after MI

Sympathetic hyperactivity is known to contribute to the pathophysiology of a variety of cardiovascular diseases, including MI [1,2]. Sympathetic ganglion neuron ion channels play an important role in sympathetic hyperactivity. Many of these ion channels are involved in the pathophysiological processes of cardiovascular diseases, such as hypertension and ischemic heart disease [22,23,24]. Blocking the Nav1.8 channel significantly attenuated ischemia-induced ventricular arrhythmia, primarily by suppressing sympathetic ganglion activity [24]. Overactivation of Cav2.2 channels in sympathetic ganglion neurons contributed to cardiac sympathetic hyperactivation and the occurrence of ventricular arrhythmogenesis [25,26]. Ion channels can serve as potential therapeutic targets in disease treatment [27]. Similarly, ion channels are the basis of neurons’ electrical activity in sympathetic ganglia. Among these channels, it has been found that sodium channels govern AP upstroke and propagation in neurons [28], potassium channels mediate the AP repolarization process [29], and calcium channels are not only involved in the process of action-potential firing but are also closely related to intracellular calcium concentration and neurotransmitter release [30,31]. These channels are also expressed in SCG neurons, and they are the basis of sympathetic nerve excitability. Our results showed that not only did the activity of neuronal ion channels increase significantly in SCG neurons after MI but the NE content in serum also increased significantly. Reducing the activity of ion channels in SCG neurons is an important way of reducing the adverse effects of sympathetic nerve overexcitation after MI. 

### 4.3. P2Y12 Expression and Roles of P2Y12 Receptor Antagonist Treatment in MI

P2Y receptors are expressed ubiquitously in the body, including in the central nervous system and microglia, with physiological roles in neurotransmission, neurogenesis, and a number of peripheral pathophysiological processes [32]. P2Y12 is a P2Y metabolic G-protein-coupled purinergic receptor expressed in sympathetic ganglia that regulates neuronal function [11]. P2Y12 is involved in the enhanced excitatory synaptic responses in the substantia gelatinosa neurons after nerve injury [12]. After MI, the expression of P2Y12 receptors in some sympathetic ganglia increased significantly [11]. In some diseases, sympathetic activity can be reduced by inhibiting P2Y12 in sympathetic ganglia [33]. It can be seen that P2Y12 is involved in the pathophysiological process of increasing sympathetic activity after MI. Downregulation of the P2Y12 receptor in the SCG after MI may improve cardiac function by alleviating the sympathoexcitatory reflex [34]. In addition, many literature reports have shown that P2Y receptor affects the characteristics of neuronal ion channels and the release of neurotransmitters [32,35,36,37]. Similarly, P2Y12 also has a direct impact on ion channels [13,14], which may be involved in the regulation of SCG neuronal ion channel characteristics after MI.

32, 6T TIC is a typical P2Y12 receptor inhibitor. TIC significantly reduced the incidence of major adverse cardiovascular events in acute coronary syndrome patients [38,39]. The application of TIC in the treatment of ST-elevated acute coronary syndrome can increase the level of myocardial microcirculation perfusion and improve left heart function [40]. TIC can protect against ischemia-induced neural injury and has neuroprotective effects [16]. In addition, TIC improved ion channel function [15] and reduced calcium influx [41,42]. Our previous studies have shown that TIC attenuated the changes in the electrophysiological characteristics of SG neurons following MI [7]. Similarly, the results of this study showed that TIC attenuated the change in the electrophysiological characteristics of SCG neurons after MI, and the expression of P2Y12 in SCGs and the NE content in serum after MI were reduced, which also confirmed the neuroprotective effects of TIC from another perspective. It can be deduced that TIC prevented the development of electrophysiological abnormalities in SCG neurons after MI by improving the expression of P2Y12 in ganglia and the characteristics of the neuronal ion channel, and it further protected the cardiovascular system and sympathetic nerves. Several studies have shown that TIC is associated with bradycardia, atrioventricular blockage, and ventricular pauses [43,44]. Caution and careful monitoring are required, especially for patients with already compromised conduction systems and/or treated with AV blocking agents [45]. The mechanism whereby TIC leads to bradyarrhythmia is unclear; one possibility is a direct effect of ticagrelor on cardiac autoregulation and conduction and another may be a modulatory effect of adenosine. TIC inhibits cellular uptake and increases the plasma concentration of adenosine [46]. However, the increase in adenosine reduces cardiac sympathetic efferent nerve activity [47]. The reduction in sympathetic nerve activity may further lead to bradycardia and atrioventricular blockage, which may be one of the mechanisms whereby TIC causes bradycardia, as is also verified from the perspective of sympathetic nerves. 

TIC is a commonly used P2Y12 receptor inhibitor in clinical contexts. In addition, it also includes prasugrel, clopidogrel, and other inhibitors. Although our study did not examine whether other inhibitors have effects on SCG neuronal ion channels after MI, the findings suggest that other inhibitors may have the same effects. No relevant research has been published at present, and the hypothesis needs to be verified in subsequent work. In conclusion, our experimental results suggest that TIC has neuroprotective effects after myocardial infarction. However, for clinical applications, ECG and sympathetic nerve activity should be monitored in patients with acute coronary syndrome when P2Y12 receptor inhibitors are prescribed. For patients with bradycardia and low sympathetic activity, P2Y12 receptor inhibitors should be used with caution. 

### 4.4. Limitations

Several limitations of this study should be recognized. Firstly, ion channel protein and P2Y12 expression were not validated by Western blotting in the present study because of the small size of the SCG tissue. Secondly, our previous studies have shown that TIC can inhibit the ion channel currents of stellate ganglion neurons, so experiments demonstrating that TIC acts on the ion channel currents of SCG neurons directly were not conducted in the present study. 

## 5. Conclusions

P2Y12 receptor antagonist (TIC) pretreatment partly reversed P2Y12 expression and abnormal neuronal electrophysiological changes in SCGs after MI. This may represent one of the mechanisms underlying the cardiovascular protection brought about by TIC.

## Data Availability

The data presented in this study are available on request from the corresponding author.

## Abbreviations

SCG	superior cervical ganglion
MI	myocardiac infarction
DAPI	4′,6-diamidino-2-phenylindole
TH	tyrosine hydroxylase
TIC	ticagrelor
ISO	isoproterenol
LAD	left atrial diameter
LVDD	left ventricular end-diastolic dimension
LVEF	left ventricular ejection fraction
FS	fractional shortening
ECG	electrocardiogram
CK	creatine kinase
CKMB	creatine kinase isoenzyme
